# Development and validation of a medication safety self-assessment tool for primary healthcare settings in China

**DOI:** 10.3389/fmed.2025.1584286

**Published:** 2025-08-05

**Authors:** Shang Yong-guang, Qin Wang-jun, Guo Dong-jie, Li Peng-mei, Zhang Lei

**Affiliations:** Department of Pharmacy, China-Japan Friendship Hospital, Beijing, China

**Keywords:** primary healthcare setting, medication safety, risk management, self-assessment, Delphi method, analytic hierarchy process

## Abstract

**Background:**

Medication safety remains a global public health challenge, particularly in resource-constrained primary care settings. This study aimed to develop and validate a specific, proactive, medication safety self-assessment tool tailored for primary healthcare settings in China.

**Methods:**

A mixed-methods approach combining literature review, field investigations, a Delphi expert consultation (3 rounds), and Analytic Hierarchy Process (AHP) was implemented. Forty-three voluntarily participating settings completed the self-assessment, with effectiveness validated through correlation analysis between assessment scores and adverse drug events (ADEs) indicators (medication errors, dispensing/prescribing errors).

**Results:**

We constructed a medication safety self-assessment tool for primary care settings comprising 5 domains, 18 core characteristics, and 84 self-assessment items. The mean percent score for 43 participating primary care settings was 81.5% ± 11.7%, and Spearman’s rank correlations were used to examine the relationship between the overall assessment scores to the incidence of ADEs (Correlation Coefficient is −0.448, *p* = 0.003).

**Conclusion:**

As the first medication safety assessment tool for Chinese primary healthcare settings, based on current medication practices and validated through ADE, implementation demonstrates potential to enhance medication safety practices.

## Introduction

Across medication treatment processes, the probability of adverse drug events (ADEs) occurring is 5.7%. Notably, primary and community healthcare settings demonstrate substantial variability in medication error rates, which span a wide range from 2 to 94% (1). According to a report from the World Health Organization (WHO), The global annual cost of medication errors is estimated at $42 billion USD (2). Considering these significant risks, “Medication Safety” was designated as the theme for World Patient Safety Day 2022. WHO Global Patient Safety Action Plan 2021–2030 calls for achieving “zero harm” in medication use and urges governments and departments worldwide to establish standardized medication safety assessment tools (MSSA) to effectively track and report on key elements based on real-world scenarios (3).

In China, primary healthcare settings (PHS) constitute a massive network, with over 1.01 million facilities undertaking 51.8% of the nation’s health care workload (4). In 2024, the National Adverse Drug Reaction Monitoring Network received 2,597,000 ADEs reports from PHS, comprising 51.2% of the total. However, lack of professionals (e.g., 1.2 pharmacists per 10,000 population), deficiency in decision-making capabilities of professionals, and shortage of infrastructural resources thereby render PHS vulnerable to medication safety risks. To systematically control the risks associated with the medication process, PHS could benefit from introducing proactive approaches, such as MSSA, to sustainably optimize medication safety in resource-constrained settings (5).

Since 2000, the MSSA has been used by individual hospitals and collaborative groups to improve safety in United States and globally (6, 7). However, this tool does not address the unique constraints of PHS. Nevertheless, due to disparities in healthcare systems and development levels between countries, in 2018 and 2020, we organized 21 tertiary first-class hospitals in China to conduct MSSA. By applying the Delphi method, the assessment items of MSSA were adapted to the Chinese healthcare and hospital settings comprising 161 assessment items. However these tools are not fully applicable to PHS in China, though it offers valuable references and insights.

This study employs a mixed-methods research design, integrating systematic literature review, field investigations, online questionnaire surveys, and expert consultations to establish a medication safety self-assessment tool in primary care settings (PMSSA). It is hypothesized that the tool’s validity could be evidenced by a negative correlation between assessment scores (high scores indicating better practices) and ADEs incidence, such that higher scores predict lower ADEs rates. The designed PMSSA aims to enable effective self-evaluation, risk identification, systematic assessment, and development of strategic interventions, thereby providing an evidence-based framework for enhancing medication safety management in PHS. Additionally, the tool can be adapted to different regional healthcare settings, allowing for tailored adjustments to local infrastructure and cultural contexts.

## Materials and methods

### Study context

The present study was carried out in Beijing, China. As the capital city, Beijing located in North China, covers an area of 16,410 square kilometers. As of 2024, it had a permanent population of 21.832 million. About 21.5% of the population was over 60 years old, and chronic diseases were relatively prevalent among the elderly (8). A vast primary care network of 2,143 PHS are spread throughout Beijing, playing a vital role in basic medical care, health management, and disease prevention for residents. In 2023, all community health service settings in Beijing provided medical services to 84.849 million patients, which accounted for 29.2% of the total number of patient visits to medical institutions across the city and represented a 31.6% increase compared to the number of patient visits in 2022 (9). Improving medication safety through self-assessment in PHS would require new tools, such as PMSSA.

### Diagram illustrating the process of PMSSA

**Figure 1:**
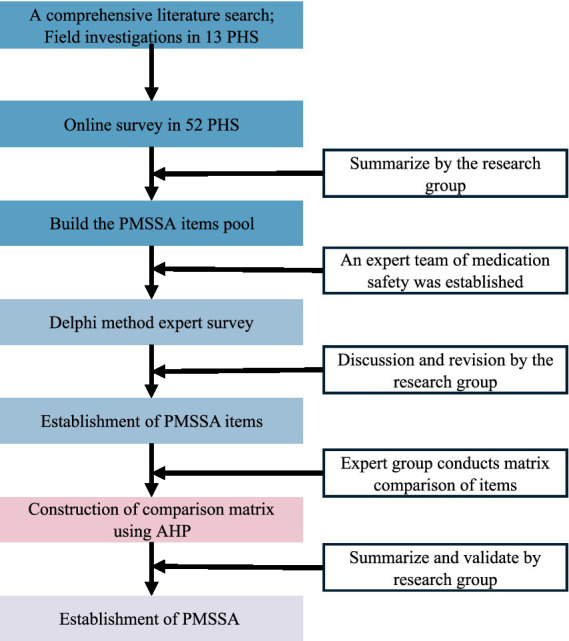
The flowchart of constructing PMSSA.

The typical risk factors of medication safety in PHS were systematically identified through literature research, in-depth field investigations, and network-questionnaires surveys. Based on these findings, the study group built the framework (core characteristics and domains) and assessment item pool of the PMSSA tool. Subsequently, the PMSSA was refined using the Delphi method for screening assessment items. Finally, AHP was employed to determine the weights assigned to these items by experts. The flowchart is visually represented in Figure 1.

### Literature research, field investigations and network-based questionnaire survey

A comprehensive literature search was conducted in major Chinese academic databases (CNKI and WANFANG) for publications up to September 2021, using “medication safety,” “safe medication use,” “ADEs,” “medication or human or dispensing or prescribing errors,” “primary healthcare institutions,” “primary care settings,” “community hospitals” as keywords. Articles with duplicates and reports not related to medication use in primary care settings were excluded. Medication safety-related issues reported in the included studies were systematically extracted, and the associated risk factors were categorized.

Field investigations were carried out at 13 PHS in Beijing, employing structured observation protocols and key informant interviews to identify critical risk factors underlying medication safety issues.

Based on these findings, a multidimensional questionnaire was developed and administered to health providers (including pharmacists, nurses, physicians, and management personnel) across 52 PHS via an online survey platform. The questionnaire comprised seven parts: 1. Scope of diagnostic and treatment practices; 2. Scope of medication therapy services; 3. Healthcare staff competency and continuing education; 4. Workforce composition and staffing patterns; 5. Medication therapy workflow and processes; 6. Implementation of health information technology and automated dispensing systems; 7. Risk management protocols and safety culture development.

#### Preliminary PMSSA

The risk factors obtained from literature review, field investigations, and questionnaire surveys were compiled and categorized using the 5M1E management model (10) (5M1E management model refers to Man, Machine, Material, Method, Measurement, and Environment) to build the preliminary framework of the PMSSA.

**Figure 2:**
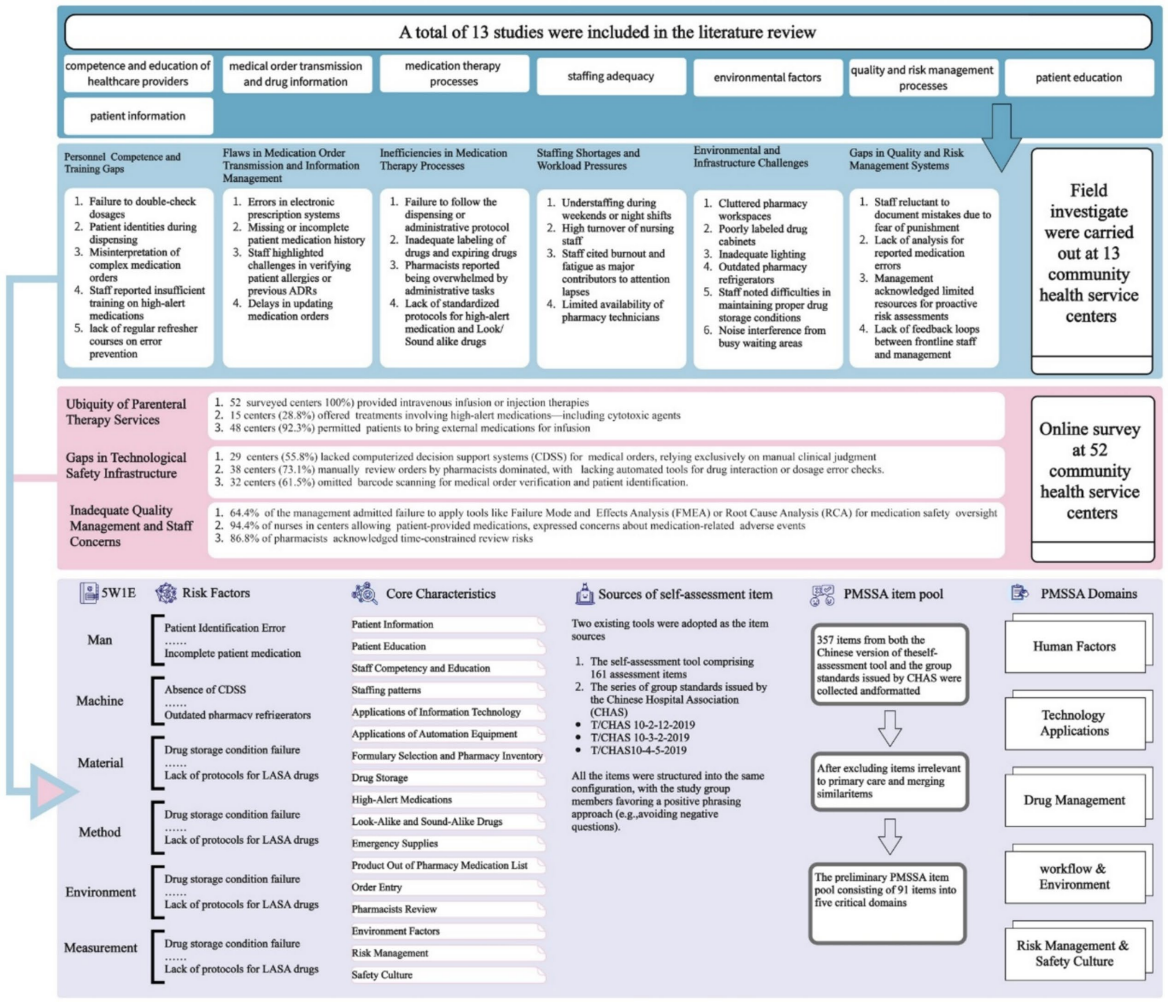
The flowchart of constructing Preliminary PMSSA.

Two item sources develop the assessment item pool. One was the Chinese version of the self-assessment tool comprising 161 assessment items. The other was the series of group standards for quality and safety management of Chinese hospitals (T/CHAS 10–2-12-2019 (11), T/CHAS 10–3-2-2019 (12), and T/CHAS 10–4-5-2019 (13)) issued by the Chinese Hospital Association (CHAS). The contents of the two selected risk management tools were combined to form items. Items concerning specialized health care, such as inpatient chemotherapy, were irrelevant to primary care settings and thus removed from the assessment pool. And items from different sources with similar meanings were integrated to form one item. The flowchart is visually represented in Figure 2.

### Delphi survey

Delphi panelists: In November 2021, a convenience sample of 15 PHS and patient safety experts based in Beijing were recruited via telephone. Eligibility criteria included: 1. At least 10 years of clinical/managerial experience; 2. Research focus or professional engagement in patient safety. All participants provided informed consent prior to enrollment.

The Delphi method is a qualitative research approach that uses multiple rounds of anonymous surveys to seek expert consensus. We implemented a three-round Delphi process following the best practice guidance on Delphi technique (14). The initial Delphi round employed a semi-structured questionnaire to collect expert feedback on the framework domains, core characteristics, and specific assessment items, as well as to evaluate item applicability. Items meeting exclusion criteria while the remaining items proceeded directly to the third round. The study team summarized the first-round results, revised or submitted new items based on expert opinions, and developed a second-round questionnaire. During the second Delphi phase, experts reassessed item applicability and provided revision suggestions. The study team systematically analyzed results and deliberated on items failing meeting exclusion criteria and revised retained items. The third Delphi round synthesized outcomes from preceding phases, establishing formal assessments of validity, importance, and feasibility.

The applicability, importance, and feasibility of assessment items were rated by experts using a 5-point Likert scale (15), with scores ranging from 5 (highest) to 1 (lowest). Consensus between experts was measured using coefficient of variation (CV), average score and full-score frequency. Full-score frequency was defined as the number of times an item was rated a full-score evaluation. Exclusion criteria for evaluating item applicability, importance, and feasibility: 1. Full-score frequency below the threshold (threshold = mean – standard deviation); 2. Average score below the threshold (threshold = mean – standard deviation); 3. Coefficient of variation above the threshold (threshold = mean + standard deviation). Candidate items meeting any one of the three criteria shall be excluded. Consensus between experts was also evaluated across all items using Kendall’s coefficient of concordance (Kendall’s *W*). If the *p*-value is less than 0.05 from the *χ2* test for Kendall’s *W*, it is inferred that the experts’ evaluation opinions show statistical consensus (16).

### AHP procedure

AHP is a multi-criteria decision-making method that is used to quantitatively analyze qualitative problems (17). We constructed decision models for the domains and core characteristics, created a comparison matrix. The experts of Delphi method will be invited to the AHP procedure, they used the Saty scale to compare the prioritize of each indicator in pairs (18).

### Application and efficiency evaluation

From October 2022 to May 2023, the research-developed assessment tool was implemented in 43 voluntary PHS in Beijing with the support of Beijing Municipal Health Commission, comprising 33 public and 10 private PHS, with annual patient volumes ranging from 50,000 to 500,000 patients. Prior to implementation, the research team conducted 4-h standardized training for core staff members from participating institutions, including: 1. Tool objectives; 2. Likert scoring principles; 3. Item-specific case studies; 4. Data submission protocols. Addressing all inquiries about the assessment tool. Each facility established a multidisciplinary assessment team (including physicians, pharmacists, nurses, and quality management personnel) to execute the evaluation. Following assessment, the collected data were submitted through a secure web-based platform^1^.

The assessment employed a five-level Likert scoring system (A-E) with corresponding numerical values (0–4 points) (19). Level A: No activity to implement recommended process; Level B: Process discussed but not implemented. Both Level A and B received 0 points. Level C: Process was partially implemented in part of the organization and received 2 points; Level D: Process was fully implemented in part of the organization and received 3 points; Level E: Process was fully implemented throughout organization and received 4 points. Results were calculated as mean percentage scores (20), where the average item score was divided by the maximum possible score (e.g., an average score of 3.20 out of 4 equaled 80%).

Dispensing errors, prescribing errors, and medication errors were selected as ADEs indicators based on medication errors were the most common ADEs. The research team analyzed medication errors from the preceding month. Dispensing errors were collected through routine quality control records (dosage, drug selection, or administration route errors); Prescribing errors were collected through pharmacist-initiated prescription modifications records; Medication errors were detected during medication reconciliation, defined as clinically inappropriate orders per institutional guidelines. Quantitative data on error frequencies were collected, and ADEs incidence rates were calculated by dividing error frequencies by total prescription volume.

### Data analysis

The data collection and analytical processes adhered to the Standards for Reporting Qualitative Research (SRQR) (21). Two researchers independently conducted dual-entry verification of expert questionnaires using Excel. Following this, a separate analyst pair evaluated the statistical interpretability of each item. Concurrent team discussions facilitated immediate data interpretation, with final conclusions undergoing systematic cross-verification to validate result consistency among all research members. Statistical analyses were performed using SPSS25 for key parameters including authority coefficient (Cr), Kendall’s coefficient of concordance (Kendall’s *W*), coefficient of variation, and correlation analyses between assessment outcomes and ADEs metrics. Weight determination employed specialized AHP-OS software.

## Results

### Literature review, field investigations and network-based questionnaire survey

A total of 13 studies were included in the literature review. The risk factors associated with medication safety issues reported in the published studies were categorized into 8 major risk factors. Based on the field investigations, the following research findings were identified and categorized according to the risk factors typically associated with medication safety in PHS, as presented in Figure 2.

A total of 571 valid questionnaires were collected from health providers across 52 community health service centers. The questionnaire survey, based on literature review and field investigations, further revealed risk factors in the medication practices of the surveyed community health service centers. The relevant core findings are summarized in Figure 2.

### Preliminary PMSSA

The risk factors were categorized using the 5M1E management model, from which 19 core characteristics of medication safety in primary care settings were derived—including Patient Information. Based on this, the team aggregated the core characteristics into five domains: *1. Human Factors; 2. Technology Application; 3. Drug Management; 4. Workflow and Environment; 5. Risk Management and Safety Culture, finally forming the preliminary framework of the PMSSA tool* (Figure 2). Three hundred and fifty seven items from both the Chinese version of the self-assessment tool and the group standards issued by CHAS were collected and formatted. After excluding items irrelevant to primary care and merging similar items, the preliminary PMSSA item pool consisting of 91 items was established, pending further Delphi refinement.

### Delphi survey

The final Delphi panel (*n* = 15 experts) consisted of 10 pharmacists, 1 nurse, 1 physician, 1 hospital information engineer, and 2 hospital quality management directors. Five experts were from primary care settings, 1 expert was from primary care management organizations, and nine experts were from general hospitals, with professional experience ranging from 16 to 38 years. All three rounds of expert consultation questionnaires were fully recovered, with a total of 124 modification suggestions. The experts have a high level of concern for the research and are actively engaged in the consultation. Authority Coefficient (Cr) was used to measure expert authority degree, which is determined by the expert’s judgment basis (Ca) and familiarity (Cs). Ca is the sum of the expert’s quantitative self-assessment scores for the four types of judgment basis (theoretical analysis, practical experience, understanding of peers, and intuition); Cs is an expert’s self-assessed level of familiarity with the research content. In this study, Cr $=\frac{\mathrm{Ca}+\mathrm{Cs}}{2}$ = 0.86, When Cr is ≥ 0.7, the expert authority degree is considered high.

In the first round of applicability evaluation, Kendall’s *W* = 0.243 (*χ2* = 62.05, *p* < 0.001). In the second round of applicability evaluation, Kendall’s *W* was 0.272 (*χ2* = 69.24, *p* < 0.001). In the third-round, the importance evaluation showed Kendall’s *W* = 0.191 (*χ2* = 48.70, p < 0.001) and the feasibility evaluation showed Kendall’s *W* = 0.180 (*χ2* = 45.83, *p* < 0.001). All results indicated statistical consensus among experts.

### First round of Delphi

All the 91 items in the preliminary PMSSA item pool were evaluated. The average applicability score of these items was 4.45, the coefficient of variation was 18.55%, and the full score frequency was 63.28%. Seven items met the exclusion criteria. Experts provided 90 modification suggestions for 37 items and 1 item supplement suggestion related to continuing education for healthcare staff. In the first round of Delphi, we submitted 1 item, merged 2 items (arrangement of working hours for daily and emergency), and revised 35 items (including the 7 items that met the exclusion criteria in this round).

### Second round of Delphi

All the 37 items revised or merged, and newly submitted items were included, The average applicability score of these items was 4.64, the coefficient of variation was 13.22%, and the full score frequency was 69.29%. A total of 34 modification suggestions were collected, leading to the revision of 31 items.

### Third round of Delphi

**Figure 3:**
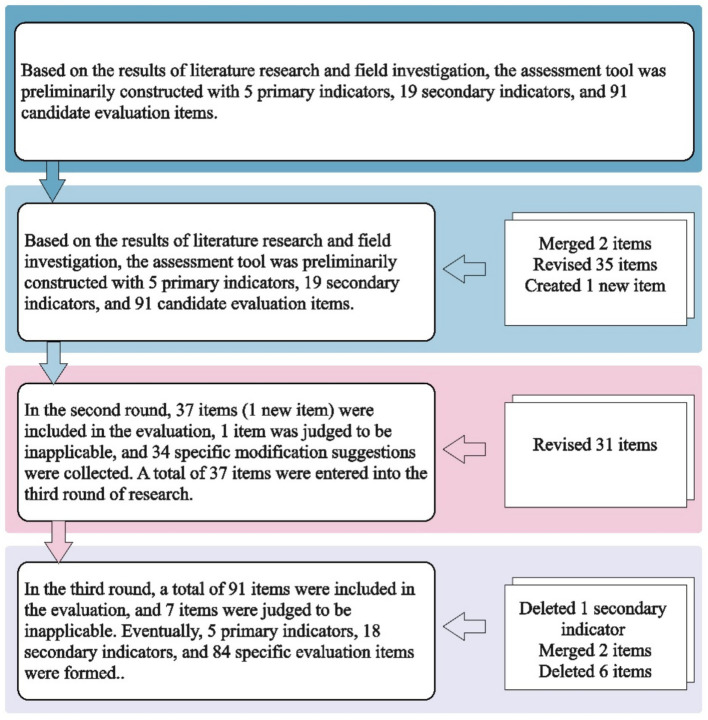
The screening process of constructing PMSSA.

Seven items were found to meet the exclusion criteria. The item“*Conducting medication safety education for staff in healthcare settings*”was retained, while the remaining 6 items were excluded. The core characteristic “*Drug Information*” was removed, and its two sub-items were merged and included in the core characteristic “*Drug Preparing, Dispensing and Administering*.” Finally, the assessment tool with 5 domains, 18 core characteristics, and 84 self-assessment items were formed. The specific items can be viewed on the medication safety self-assessment website (see footnote 1). Figure 3 describes the screening process of assessment item in detail.

### Results of determining the weights using the AHP method

We constructed a hierarchical analysis structure comprising 5 domains and 18 core characteristics, developed judgment matrices for various hierarchical indicators, and obtained an eigenvalue of 5.214 for the domains via AHP calculations, with the Consistency Ratio (CR) value of 0.048. The CR values of all 18 core characteristics were below 0.10, indicating excellent consistency in the judgment matrix. Detailed results can be found in Table 1.

**Table 1:** Weighting of the domains and core characteristic of the medication safety assessment.

Domains	Core Characteristic	Number of self-assessment item	Combination Weight	maximum Eigen value	CR values
1. Human factor	1.1 Patient information	3	7.75%	4.123	0.045
	1.2 Patient education	5	3.12%		
	1.3 Staff competency and education	7	11.73%		
	1.4 Staffing patterns	4	11.73%		
2. Technology applications	2.1 Information technology applications	8	8.87%	2.000	0.000
	2.2 Automation equipment	3	4.43%		
3. Drug management	3.1 Product selection and pharmacy inventory	6	8.80%	6.282	0.045
	3.2 Drug storage	5	4.88%		
	3.4 High-alert medications	3	3.35%		
	3.5 Product with similar/confusing packaging and look/sound alike name	4	3.01%		
	3.6 Emergency supplies	3	2.63%		
	3.7 Product out of pharmacy medication list	3	1.22%		
4. Workflow and environment	4.1 Order Entry	6	5.23%	4.061	0.022
	4.2 Pharmacists review	4	3.08%		
	4.3 Drug preparing, dispensing and administering	5	3.08%		
	4.4 Environment Factors	3	0.93%		
5. Risk management and safety culture	5.1 Risk management	6	12.08%	2.000	0.000
	5.2 Safety culture	6	4.03%		

### Application results and efficiency evaluation of PMSSA

**Figure 4:**
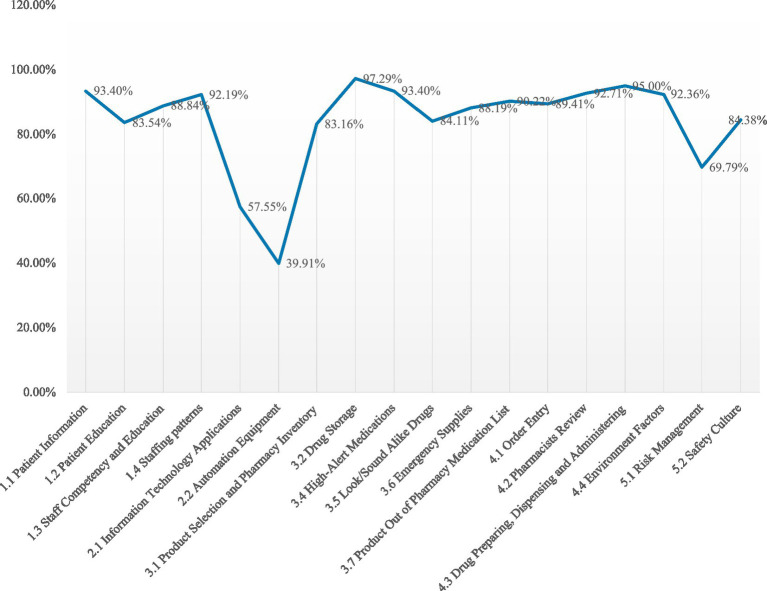
The mean percent score for core characteristics.

The overall achievement rate for 43 participating PHS was 81.5 ± 11.7%. The mean percent score for domains: *“Human Factors”* at 87.0%, *“Technology Applications”* at 47.1%, *“Drug Management”* at 84.6%, *“Workflow and Environment”* at 88.0% and *“Risk Management and Safety Culture”* at 71.9%. The mean percent score for core characteristics were described in Figure 4.

**Figure 5:**
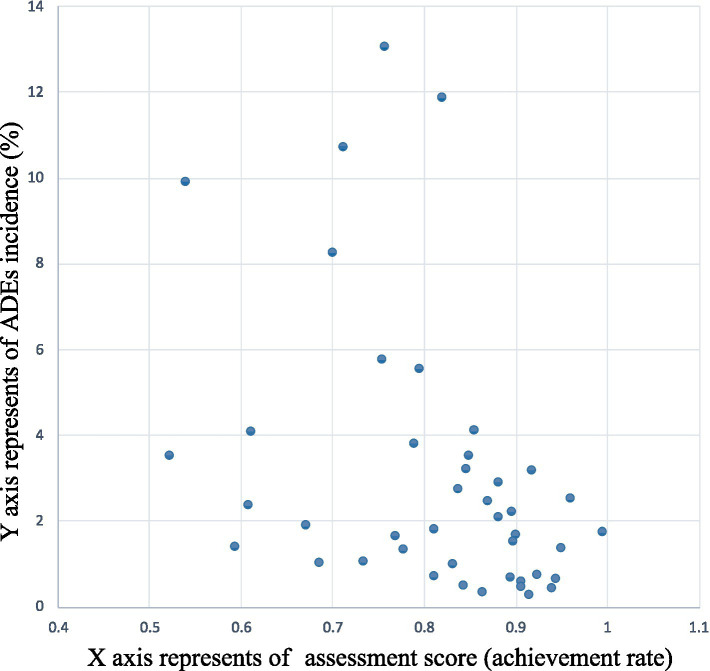
The correlation between the incidence of ADEs and the assessment scores.

Incidence of the dispensing errors was 0.14% (95% CI 0.07 to 0.21), prescribing errors was 2.34% (95% CI 1.52 to 3.15) and medication errors was 0.58% (95% CI 0.30 to 0.86) in 43 participating PHS. The sum of the three terms as incidence was 3.06% (95% CI 2.08 to 4.04) as the overall ADEs incidence. The correlation between the ADEs incidence and the assessment scores in participating PHS was examined using Spearman’s correlation analysis, yielding a correlation coefficient of −0.448 (*p* = 0.003, *N* = 43). The results are shown in Figure 5.

## Discussion

### The reliability of comprehensive research methods

The Delphi method is a highly regarded approach to group decision-making that relies on the collective theoretical and practical expertise of respondents. By soliciting anonymous opinions from experts, this method effectively protects them from being influenced by authoritative figures. Through repeated information exchange and feedback, the Delphi method achieves a comprehensive consensus among experts, making it widely used in the establishment indicator systems (5, 19). The Analytic Hierarchy Process (AHP) decompose complex decision-making problems into hierarchical structures, suitable for determining the priority of evaluation indicators across levels (22). Weighting results are verified through logical consistency tests, ensuring that experts’ judgments on indicator importance are logically consistent. This establishes a scientific and rigorous weighting system.

We saw another group describes their experiences and findings using a combination of a Delphi approach, complemented by item weight and performance weight decision-making principles (23). Their questionnaire is based on the 2011 ISMP Medication Safety Self-Assessment, whereas our PMSSA were developed via literature research, on-site visits, and questionnaire surveys conducted. The experts selected by our team covered key domains of medication safety, were familiar with primary care, and showed high authority and enthusiasm. Due to the varying focuses and understandings of medication safety among experts, 124 revisions to the candidate items were proposed. Based on the expert evaluation, the items were quantitatively screened using the threshold method. By using stringent exclusion criteria, we aim to retain key risk factors and establish a PMSSA for PHS.

We have successfully validated the efficacy of the assessment tool and discovered a significant negative correlation between the incidence of ADEs and the assessment results. This finding suggests that the assessment results effectively reflect the current of medication safety status in PHS.

### The characteristics of our self-assessment tool

Clear Structure: Adopting the classic 5M1E management model*, —“Man, Machine, Material, Method, Measurement, and Environment”*—the assessment tool provides a 5-domains comprehensive indicator framework.

Risk-Focused items: Based on research on drug-use risk factors in PHS, each domain of PMSSA covers corresponding core characteristics. For example, the domain *“Drug Management”* includes seven core characteristics—*"Product Selection and Pharmacy Inventory,” “Drug Storage,” “High-Alert Medications,” “Emergency Supplies,” “Look-Alike/Sound-Alike Drugs,”* and *“Product Out of Pharmacy Medication List”*—which were derived from key risk factors across the *entire* drug management process. Using 19 core characteristics derived from critical risk factors, 84 risk-focused items were developed through screening and revision via the Delphi method.

Compact and Comprehensive: With 84 items, the tool effectively covers the primary risk factors associated with drug use in healthcare settings, enhancing the enthusiasm and efficiency of self-evaluation among primary healthcare providers.

Applicable and Feasible: The final included items demonstrate to be relevant and practical. Under the domain *“Workflow and Environment,”* the core characteristic *“Drug Preparing, Dispensing and Administering”* comprises two assessment items: A. Before medication administering, drug treatment, medical staff should inspect the drug’s appearance and expiration date, and verify the drug name, administration time, dosage, frequency, and administration route according to the prescription or medical order. B. Prior to administration, health providers should routinely explain the drug’s name and purpose to the patient or their family members. Before the first dose, staff must confirm the patient’s drug allergy history and inform them of potential adverse reactions and precautions. The two evaluation items integrate *“drug dispensing”* and *“patient education”* to achieve drug double checking, rather than merely emphasizing “double checking” and “enhanced labeling” (24, 25). This approach aligns with the operational realities of China’s primary healthcare system, promoting both safety and efficiency in clinical practice.

### Results of the preliminary assessment

The low scores in *“Technology Applications”* (47.1%) included two core characteristics: *“information technology application”* and *“automated equipment”* use scored merely 57.55 and 39.91% among the 43 participating settings. In recent years, there has been a consistent enhancement in the informatization and automation levels of medical settings in China. Notably, primary healthcare settings have progressively integrated advanced automated equipment such as fully automated integrated dispensing machines and intelligent medicine cabinets. However, the evaluation results revealed that these PHS generally failed to incorporate risk management concepts into automated equipment applications. Additionally, studies have demonstrated that computerized physician order entry (CPOE) systems and clinical decision support systems (CDSS) can reduce medication errors and unreasonable prescriptions (26). By contrast, our assessment showed that the existing information systems lacked warning and assistant review functions for unreasonable physician orders, with prescription creation and review relying entirely on manual operations.

### Study limitations and strengths

The limitations of validity and reliability of qualitative research have always existed, as criteria for determining the trustworthiness of qualitative research have not been examined (27). However, to ensure research credibility, we developed PMSSA tool based on real-world settings and we adhered to Delphi survey guidelines, including expert and monitor selection, Delphi method steps, and avoidance of authority bias. Data from literature reviews, on-site visit records, online surveys, and expert feedback can be revisited as needed.

Another limitation is the implementation of the tool and the assessment results. The implementation of PMSSA crucially depends on a constructive organizational culture and supportive hospital management. Fortunately, our study was supported by Beijing Municipal Health Commission, ensuring the participation enthusiasm of PHS. The tool requires validation in diverse PHS to account for variability in staffing models and medical infrastructure. It is necessary to iterate and add optional terms and weight calculation algorithms to enhance generalizability. The assessment results were based on the subjective judgment of the participants. Self-assessment may overestimate compliance due to social desirability bias. To ensure both standardized assessment implementation and objective result interpretation, we conducted training for multidisciplinary teams of participating facilities on assessment significance and scoring criteria and collected assessment data via a secure web-based platform. Participating facilities could only view their own results and averages; yet we must acknowledge that subjective evaluation is not solely determined by true value—individuals may interpret identical facts differently (28). Future iterations should incorporate observational audits to mitigate potential biases.

## Conclusion

Building upon a comprehensive analysis of the current medication safety landscape in PHS, the study team developed and validated the PMSSA. Although the subjective and self-evaluation methodology may introduce potential limitations in result interpretation and validity generalization, it represents a practical contribution to heightening awareness of key features of safe medication system for PHS and could serve as a practical tool to help them enhance medication safety. The systematic development process, combined with preliminary validation findings, offers valuable methodological insights and practical reference points for healthcare professionals and researchers working in medication safety field.

## Data Availability

The original contributions presented in the study are included in the article/supplementary material, further inquiries can be directed to the corresponding author.

## Footnotes

^1^ http://118.195.241.80:7002/
